# Tafenoquine: A 2018 Novel FDA-Approved Prodrug for the Radical Cure of *Plasmodium vivax* Malaria and Prophylaxis of Malaria

**DOI:** 10.3390/ph12030115

**Published:** 2019-07-30

**Authors:** Annie Mayence, Jean Jacques Vanden Eynde

**Affiliations:** 1 Haute Ecole Provinciale de Hainaut-Condorcet, 7330 Saint-Ghislain, Belgium; 2 Formerly head of the Department of Organic Chemistry (FS), University of Mons-UMONS, 7000 Mons, Belgium

**Keywords:** aminoquinoline, approved drugs, FDA, hypnozoite, malaria, *Plasmodium*

## Abstract

Tafenoquine (an 8-aminoquinoline) was approved by the Food and Drug Administration (FDA) in 2018 for the radical cure of *Plasmodium vivax* malaria and preventive action against malaria. Despite the fact that the mechanism of action of the drug remains unclear, all studies indicated that a metabolite is responsible for its efficacy. Routes for the preparation of the drug are described.

## 1. Introduction

Despite many campaigns of prevention and vaccination, malaria remains a major cause of deceases in developing countries. Following the World Malaria Report 2018 [1], published by the World Health Organization (WHO), the disease still affected 219 million people and killed 435,000 patients in 2017. The infection is caused by protozoans named *Plasmodium*, whose life cycle has been elegantly illustrated by Campo et al. [2] Five species are responsible for the human disease: *P. falciparum*, *P. vivax*, *P. ovale*, *P. malariae*, and *P. knowlesi*. 

**Figure 1:**
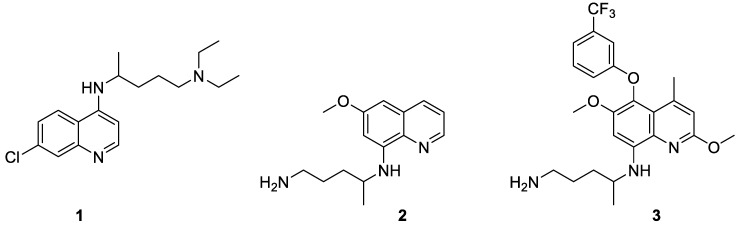
Structure of aminoquinolines (**1**–**3**).

Chloroquine (**1**, Figure 1), a 4-aminoquinoline, still constitutes a common, cheap, and safe drug for the treatment of malaria caused by any type of *Plasmodium*. However, its intensive use has led to the emergence of resistant strains of parasites. Actually, it is recommended to fight those resistant strains by administration of two drugs with different modes of action. Currently, Artemisinin-based Combination Therapy (ACT) is the most popular treatment [3].

It is worth noting that, contrary to the other species of *Plasmodium*, *P. vivax* and *P. ovale* produce hypnozoites during their life cycle in the liver. They are latent forms of the parasite, which are able to reactivate the illness, a relapse, without any bite of an infected mosquito. Primaquine (**2**, Figure 1; Primacin™), an 8-aminoquinoline, is the drug of choice to eradicate hypnozoites and is often used in conjunction with other anti-*Plasmodium* agents (schizontocides) in the radical cure of vivax malaria [2,3,4]. Tafenoquine (**3**, Figure 1), another 8-aminoquinoline, was discovered by the Walter Reed Army Institute of Research (WR238605) in 1978. After numerous in vitro and in vivo studies, it has been shown to be superior to primaquine as a novel anti-malarial agent. More details on tafenoquine are given below.

## 2. Tafenoquine

### 2.1. Names

The International Union of Pure and Applied Chemistry (IUPAC) name of tafenoquine is 4-*N*-[2,6-dimethoxy-4-methyl-5-[3-(trifluoromethyl)phenoxy]quinolin-8-yl]pentane-1,4-diamine, CAS 106635-80-7.

Tafenoquine (**3**) is commercialized, as the succinate salt (CAS 106635-81-8) of racemate, under the names Krintafel ™ [5] (tablets of 150 mg) by GlaxoSmithKline (GSK, Brentford, Middlesex, UK) as well as Arakoda ™ [6] and Kodatef^®^ (tablets of 100 mg) by 60 Degrees Pharmaceuticals LLC (Washington, DC, USA).

### 2.2. Uses

Tafenoquine was approved by the US Food and Drug Administration (FDA) on 20 July 2018, for radical cure of *Plasmodium vivax* malaria (Krintafel™), that indication is part of the portfolio of GSK. The drug is also prescribed for the prophylaxis of malaria. That aspect is handled by 60 Degrees Pharmaceuticals LLC.

### 2.3. Mechanisms of Action

**Figure 2:**
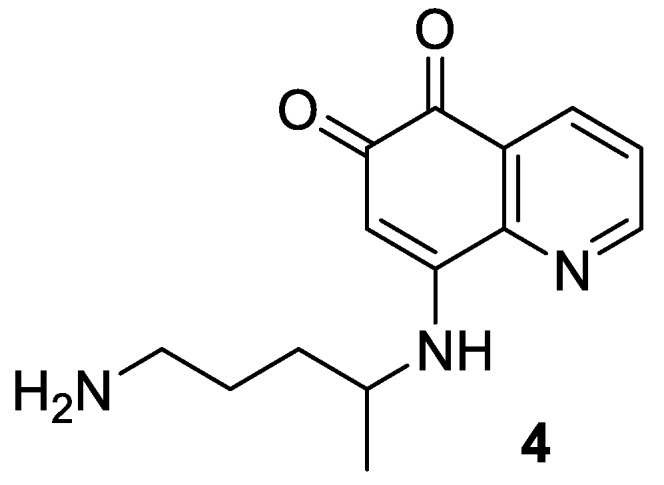
Key metabolite (**4**) of 8-aminoquinolines **2** and **3**.

There remain some uncertainties concerning the mechanism of action of 8-aminoquinolines in general and tafenoquine in particular. The radical cure activity of primaquine (**2**) and tafenoquine (**3**) is thought to be related to the 5,6-quinone species **4** (Figure 2), one of the numerous metabolites [7] produced through degradation by cytochrome P450 2D6 liver microsomal enzyme. That hypothesis was founded on in vitro [8] and in vivo [9] studies. In addition, the *ortho*-quinone **4** was detected during a pharmacokinetic analysis performed from animals that had received 8-aminoquinolines [10]. The same quinone (**4**) could be responsible for adverse effects (hemolytic anemia) in patients with glucose-6-phosphate dehydrogenase (G6PD) deficiency, leading to a contraindication for pregnant women [2,11].

Interestingly, the presence of the aryl ether in position 5 of tafenoquine **3** decreases the susceptibility of oxidation required to yield **4** [2]. Not surprisingly, an elimination half-life of 14 days has been observed for tafenoquine **3** against a few hours for primaquine **2** [12]. Consequently, comparable anti-plasmodial efficacy can be attained in humans with a single dose only of 300 mg of **3**, whereas primaquine (**2**) must be administered daily (15 mg) for two weeks [2].

### 2.4. Clinical Studies

As early as 1998, tafenoquine **3** was administered, under the control of the Walter Reed Army Institute of Research, to 48 healthy adult men in order to evaluate its safety and efficacy and to collect pharmacokinetics data [13]. Later on, GSK sponsored the first clinical trial recorded in the National Institutes of Health (NIH) data [14] for tafenoquine. It was initiated in March 2009 (NCT00871156) under the official title “Safety, Tolerability, and Pharmacokinetic Study of Concomitant Chloroquine and Tafenoquine in Healthy Volunteers”. It was a phase 1 study involving 68 participants and was described in the paper of Miller et al. [15] in 2013. The trial has been followed by five other completed phase 1 studies, five completed phase 2 studies, and only one [16] completed phase 3 study. Hemolysis risks (NCT01205178 and NCT02216123) and retinal effects (NCT02658435) were among specific side-effects targeted in those investigations. Both companies commercializing tafenoquine (GlaxoSmithKline and 60 Degrees Pharmaceuticals LLC) are actually recruiting volunteers for two phase 2 studies (NCT03320174 and NCT02563496) and one phase 3 study (NCT02802501).

### 2.5. Syntheses

**Scheme 1:**
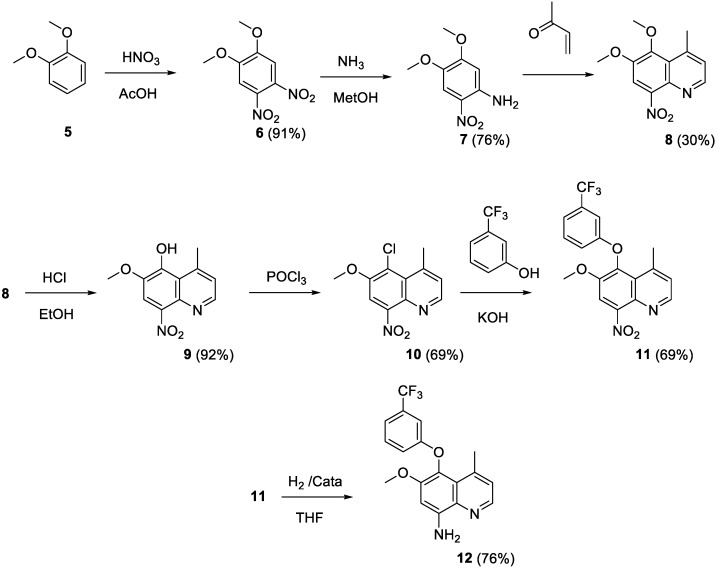
Preparation of compound **12** from veratrol.

Historically, the key intermediate in the preparation of tafenoquine is 4-methyl-5-(3-trifluoromethylphenoxy)-6-methoxy-8-aminoquinoline **12** (Scheme 1). Two alternate routes have been described to access that derivative.

One route (Scheme 1) started from veratrol (**5**) [17]. It was nitrated in positions 4 and 5. One nitro group was reduced by treatment with ammonia in methanol and the quinoline system **8** was built by a Skraup reaction involving methyl vinyl ketone. The 5-methoxy group was selectively demethylated and the hydroxy derivative was converted to the 5-chloroquinoline **10** by phosphorus oxychloride. Subsequent action of 3-hydroxybenzotrifluoride and reduction of the 8-nitro group afforded the key intermediate **12** in an overall yield of 7%.

**Scheme 2:**
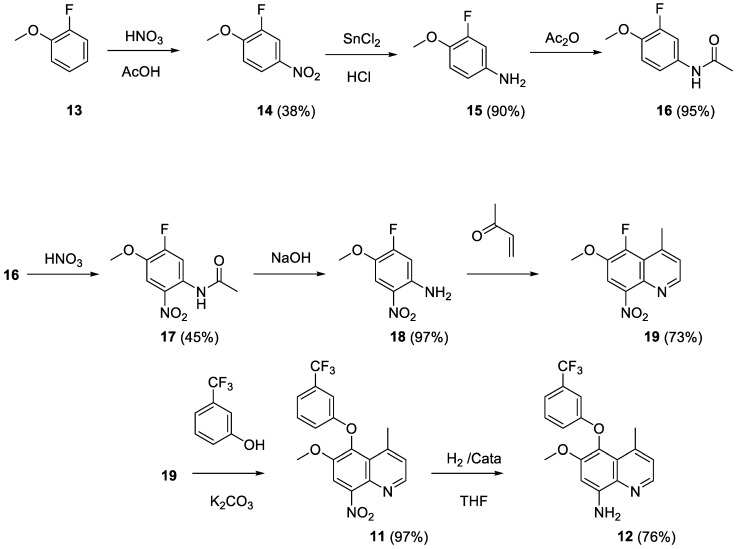
Preparation of compound **12** from 2-fluoroanisole.

The alternate route (Scheme 2) started from 2-fluoroanisole (**13**) [17]. The compound was nitrated and the nitro group was reduced to the aniline **15**. Deactivation of the amino function was achieved by acetylation allowing nitration in position 5. Deacetylation of **17** followed by the Skraup reaction afforded the 5-fluoroquinoline **19**. Fluorine displacement by 3-hydroxybenzotrifluoride followed by reduction of the 8-nitro group yielded **12** with 8% overall yield.

**Scheme 3:**
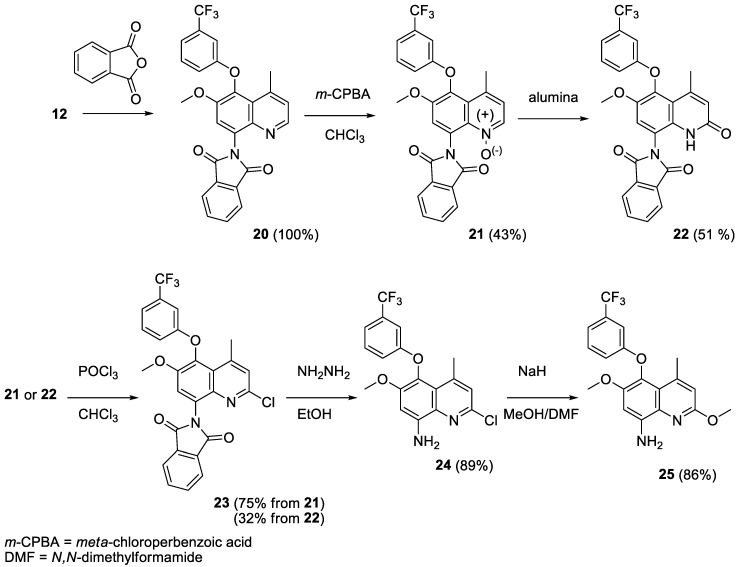
Preparation of the 8-aminoquinoline **25**.

In order to introduce the second methoxy moiety in position 2 (Scheme 3), the 8-amino group was protected by conversion into the phthalimido compound **20**. Formation of the quinoline N-oxide **21** enabled chlorination in position 2 of the fused heterocyclic system, eventually through the quinolone **22**. After deprotection of the 8-amino function, treatment with sodium hydride and methanol (or sodium methoxide) yielded the 2-methoxy derivative **25** [18].

**Scheme 4:**
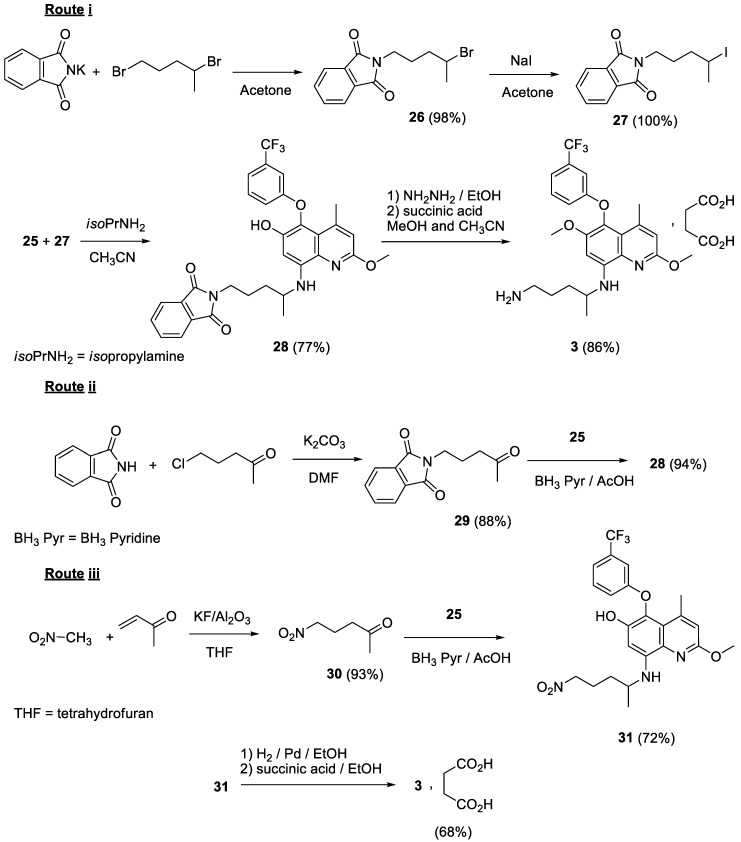
Three strategies yielding tafenoquine **3** from the 8-aminoquinoline **25**.

Finally, tafenoquine **3** has been obtained (Scheme 4) by alkylation of compound **25** through three different routes involving (i) action of *N*-(4-iodopentyl)phthalimide and subsequent hydrazinolysis of the phthalimido group [18,19,20]; (ii) reductive amination by *N*-(4-oxopentyl)phthalimide and subsequent hydrazinolysis [21]; or (iii) reductive amination by 5-nitropentan-2-one followed by reduction of the nitro group [21]. Treatment of **3**, isolated or not, with succinic acid afforded the commercial succinate salt of tafenoquine.

## 3. Perspectives

Mention should be made that, contrary to primaquine, tafenoquine **3** can inhibit hematin polymerization and therefore can exhibit, in addition to its hypnozoiticidal action, an antiplasmodial activity during the blood stage of the parasite [22]. Consequently, several clinical trials are dedicated to evaluations of tafenoquine, alone or in combinations, for malaria prophylaxis [23] and treatment of *P. falciparum* parasitaemia (NCT02488902, NCT02488980, and NCT02491606).
